# Professional Duties and Practical Suggestions

**Published:** 1884-06

**Authors:** Charles E. Francis

**Affiliations:** New York


### THE DENTAL REGISTER.

VOL. XXXVIII.] JUNE, 1884. [No. 6.

### COMMUNICATIONS.

### Professional Duties and Practical Suggestions.

Read before the New York State Dental Society.

BY CHARLES E. FRANCIS, D.D.S., M.D.S., NEW YORK.

Securing to members of the human family their organs of mastication in as perfect condition as possible through the period of their earthly pilgrimage is the highest aim and most earnest desire of the dental surgeon.

This, to be successfully accomplished in these times of physical degeneracy, where exalted types of civilization and refined culture sway both public and individual minds—where hygienic laws are lost sight of in morbid desires to gratify acquired tastes and sensual caprices; or a pampering to habits of extravagance that arbitrary fashion has created, will often require a degree of wisdom and manipulative skill which comparatively few individuals naturally possess.

Even though fitted for their calling by systematic educational training, and favored with opportunities afforded by an extended experience, many of our specialty are sometimes sorely perplexed with difficult or complicated cases which come to them for treatment, and not unfrequently are they disheartened to meet with failures when their best efforts have been expended in hopes for success.

To say that a dentist's life is "smooth sailing," and his duties "simple and light," is to express an opinion as absurd as it is erroneous. On the contrary, it is a life of perpetual anxiety and constant strife. If faithful to his calling and the best interests of his patients, his chief and ardent desire, the dentist will devote himself assiduously to his duties and improve every opportunity for increasing his store of professional knowledge, that by intelligence thus gained he may be enabled to more perfectly perform his operations; more clearly foresee contingencies, and best determine just what course of treatment will, in the diversity of cases presented, insure the most satisfactory results.

It is not always wise to jump at conclusions hastily, or to decide important matters with little or no consideration. An error of judgment; an opportunity lost or unimproved; or even an operation improperly managed may be the cause of serious trouble and perhaps irreparable mischief.

It is evident that the progressive members of our fraternity fully recognize the need and value of the highest order of dental service to the public, and how dependent all civilized communities are upon such service for comfort, health, and longevity. The fact of such recognition is demonstrated by the efforts constantly being made for improvement in all departments of dental practice and the earnest labors for educational advancement. It is also evident that the spirit of progress is more clearly manifested as each successive year rolls on, yet, to the ambitious, the goal of professional excellence is still in the distance.

In efforts towards advancement there is a tendency on the part of many of us to bend our energies in some particular direction, failing to look about for the purpose of seeking broader or safer ways which might lead to the point we are striving to reach. We sometimes seem to imagine that there is but one fixed course to pursue, so with this single idea in mind we boldly press forward, overcoming as best we can whatever obstacles may beset our path, yet not without making some misstep or beating some inglorious retreat.

Individual members of our specialty in their professional deliberations wander in different directions, exhibiting diversities of opinion in regard to methods of practice, and the treatment of special cases brought before them. Particularly is this noticeable at our associate gatherings where varied and antagonistic opinions are expressed with such decision, and advocated with so much force that many a hesitating listener is seriously perplexed to decide who are the wisest counsellors, or what methods are safest to adopt. Of course it is not to be supposed that all people can think alike or entertain a common opinion in regard to all things; but it is not necessary to fortify ourselves with ultra ideas and dogmatic notions as surrounding safeguards, in order to avoid accepting theories of others which do not favorably impress us; neither is it fair to intimate that they who differ with us are devoid of sense. From the suggestions of our co-workers may often be gathered many grains of wisdom, even though in some instances their statements require considerable sifting. Ultraism is unbridled in its tendencies and apt to prove unsafe. Better that we be governed by circumstances and the modified influences, occasions suggest.

As has already been stated, the highest ambition of the dentist in his official capacity is to insure to his patients as many of their natural teeth as possible and keep them in a condition of health and usefulness. This often involves the exercise of good judgment, great care, and fine artistic skill, together with the ability to proffer the most sensible advice that can be bestowed upon patients for their daily consideration. Of all ages and in different conditions of health, do patients come to us for advice and treatment, and of course must be considered and treated in the condition in which they present themselves. Some of them are splendid specimens of the "genus homo," robust and full of physical vigor, having inherited from a race of hardy ancestors constitutions more valuable to them than anything possible for wealth to procure. In such cases we may expect to find the organs of mastication exceedingly compact in structure, displaying a coating of enamel of adamantine hardness. Such teeth are easily cared for and ordinarily require little attention on the part of the dentist, although cases are not uncommon where teeth perfect in arrangement, as also in form, color, and texture, have, from habitual neglect, become so loaded with incrustations of calculus and other accumulations as to seriously impair their integrity and lessen by many years their period of usefulness.

But individuals who are blessed with perfect dentures are by no means the class who usually visit the dentist for treatment.

They who fill our chairs are not so greatly favored. Children are brought to us who even before three summers of their lives are completed have experienced the tortures of odontalgia and their teeth found defective at many points. Patients come to us with teeth in various stages of decay; teeth badly worn, jagged, or much broken away; teeth with enamel abraded or partly decalcified; teeth girdled with caries, or perhaps reduced to mere crownless roots. They come to us with sickly dental pulps, or pulps devoid of every spark of vitality. They come driven by the sharp, merciless agony that indicates pulpitis, or the deep throbbing pain that peridental inflammation occasions. We have also numberless cases of alveolar abscess and facial neuralgia; alveolar absorption and exostosis; epulis, fungi, and other abnormities. We have to deal with dental irregularities of every sort and grade; with contracted dental arches where teeth are sadly crowded together, distorted, deranged, and much out of normal position. Teeth too, exceedingly incorrect in articulation, with perhaps the inferior maxilla dwarfed and the under teeth far inside the line of the upper ones forcing the latter forward with such frightful prominence as to prevent the lips from closing together. Cases also where the inferior maxilla is elongated or over-developed and the under teeth projecting far outside the upper ones, giving the chin undue and ungraceful promnence. Cases are quite common where in second dentition the teeth are found decayed almost as soon as erupted—teeth which from ill-health, or some systemic disturbance during the period of their calcification, were malformed or impoverished. Also teeth dotted with numerous enamel pits or marred by deep open sulci showing enamel fusions incomplete and imperfect. And even where teeth are passably well calcified, lengthy periods of sickness, and neglect to care for them, may result in their ruin. We are daily confronted with patients whose teeth bear evidence of unpardonable neglect; the interstices of which are found loaded with extraneous accumulations; or the enamel stamped with dark, repulsive crescent shaped stains along their labial and buccal borders.

Notwithstanding the many teachings and admonitions on the part of our fraternity, the majority of people in all communities are habitually careless or negligent in the care of their masticators, and as a consequence compartively few individuals witness the anniversary of their twentieth birthday with perfect dentures, while many lose half their complement before this period of life is reached.

For the management of the many cases that come within the province of our specialty, no fixed rule can be stated that will answer for them all, yet certain principles exist which, if rightly understood and intelligently applied, would greatly aid us in our efforts to equal each occasion, and encourage us to hope for good results.

The first duty we owe our patients is to impress upon their minds the necessity of keeping their masticators in a condition of cleanliness. It has been written that "Cleanliness is next to Godliness." Certainly, with the teeth, it is their only salvation. People usually shirk this important duty. Some few cleanse them with an approximate degree of thoroughness, while many do it as if a disagreeable task which they are inclined to hurry through with as quickly as possible, and perhaps do this little at times when the least benefit is gained from the operation. Very few individuals know how to manipulate a tooth-brush skillfully or effectively. A few rapid horizontal dashes and a possible "promise," but nothing more. A tooth-brush should be used much as if the bristles were a bundle of tiny tooth-picks, and be patted gently against the teeth on all sides with a view of freeing the interstices from extraneous collections. This should be followed by much washing and rinsing with tepid water. A simple dentifrice may also be employed to advantage, and if injury from acidulated secretions be observed, liquor calcis, castile soap, or some other mild alkali may be used. Were it not for the fact that proportionately so few individuals understand at what times during the twenty-four hours of each day their teeth most need brushing, it would seem almost needless to state that our patients should be instructed to unfailingly cleanse their teeth prior to retiring at night; and they should know the evil consequences of neglecting this duty.

Parents should be made to comprehend the importance of giving their children's teeth attention at an early age, and of keeping their deciduous teeth preserved until time for their successors to appear. If suffered to decay, their usefulness is destroyed and digestion is impaired. The pain they occasion results in loss of sleep and an undue strain upon the nervous organism. If prematurely removed, the alveolar border is disturbed and in some degree arrested in its development, the dental arch becomes contracted and the new teeth are crowded out of their natural position. Irregularities thus occasioned should be timely corrected. Sometimes the result is best secured by the removal of two or four teeth, but more frequently regulating appliances are required to expand the dental arches and draw the teeth into line. Overcrowded teeth are liable to decay on their proximate surfaces and require relief, and especially so where unmistakable evidence of dissolution are visible. Twenty-eight good sound teeth are of infinitely greater value than thirty-two that are defective and loaded with filling. In endeavoring to save too many, sometimes too many are lost.

Where extraction is decided upon, the most defective teeth back of the canines should be sacrificed. The six year molars are usually the most impoverished and first to give out.

Our patients should be taught the importance of having their teeth examined at somewhat frequent intervals and before caries or calculus can have caused much mischief. The "stitch-in-time" principal is as applicable to teeth as to anything else.

In preparing cavities for the introduction of fillings, thoroughness should be conscientiously observed. Care, of course, is necessary, to avoid wounding pulps, but the walls and margins of a cavity cannot be too nicely prepared. If kept dry during the process of preparation they can be excavated with more safety, greater rapidity, and less pain to the patient; hence the rubber dam will in many cases prove exceedingly serviceable.

As regards the importance of keeping cavities dry when introducing stoppings, it seems hardly necessary at this period of dental progress to intimate that moisture should be carefully excluded in all cases, regardless of the material used for this purpose.

In a choice of material for filling cavities, a more than ordinary degree of good judgment is sometimes requisite. The age of the patient, the condition of health, quality of tooth-structure, location of cavity, and other things are to be considered.

Cavities should be filled with whatever material will, under the circumstances and peculiar condition of each case, best keep them. As a rule, gold is unquestionably the most suitable material employed, but in some instances other fillings serve a better purpose.

For deciduous teeth, plastic fillings are advisable. Gutta-percha fillings, if well impacted, seal cavities quite perfectly and will keep them in a state of preservation from two to ten or more years without changing, depending, however, much on the location of cavities and exposure to attrition. It is easly introduced and can be readly replenished when worn away. Oxy-phosphate of zinc will in some mouths keep nicely for several years, but as a rule needs frequent replenishing. For frail walls and badly broken teeth it answers a fair purpose and if occasionally examined and kept in repair will prove quite serviceable. In using this material in approximal cavities extending quite to the gum, it is safer to line cervical walls with gutta-percha stopping. Nicely fitted gold caps or crowns securely anchored to large zinc fillings will prevent them from wearing away.

As regards the use of amalgam—probably more has been said for and against it than of any or all other fillings. Most of us have witnessed cases where it has perfectly preserved cavities from further decay for many years, yet sometimes it recedes from the marginal walls of cavities in which it is impacted and this constitutes the most serious objection to its use.

Gentleman, a paper touching upon practical points pertaining to dentistry could be drawn to a wearisome or almost endless length. Not wishing to overtax your patience we will only add, that trials and perplexities will ever beset the dentist in his endeavors to fulfill the duties of his vocation. No matter how competent or skilled he may be, occasional disapointments will surely come, for infallibility is not a human attainment. And even though in some instances our best efforts are illy appreciated or poorly rewarded, let us be faithful to our trusts and so sustain the good reputation our profession has struggled to gain.

